# *Aeromonas sobria* Serine Protease Degrades Several Protein Components of Tight Junctions and Assists Bacterial Translocation Across the T84 Monolayer

**DOI:** 10.3389/fcimb.2022.824547

**Published:** 2022-02-22

**Authors:** Mitsunobu Ueda, Hidetomo Kobayashi, Soshi Seike, Eizo Takahashi, Keinosuke Okamoto, Hiroyasu Yamanaka

**Affiliations:** ^1^ Laboratory of Molecular Microbiological Science, Faculty of Pharmaceutical Sciences, Hiroshima International University, Kure, Japan; ^2^ Laboratory of Medical Microbiology, Department of Health Pharmacy, Yokohama University of Pharmacy, Yokohama, Japan; ^3^ Collaborative Research Center of Okayama University for Infectious Diseases in India, National Institute of Cholera Enteric Diseases, Kolkata, India

**Keywords:** *Aeromonas sobria*, serine protease, intestinal epithelial barrier, tight junction, claudins, ZO protein, bacterial translocation

## Abstract

*Aeromonas sobria* is a Gram-negative pathogen that causes food-borne illness. In immunocompromised patients and the elderly, *A. sobria* opportunistically leads to severe extraintestinal diseases including sepsis, peritonitis, and meningitis. If *A. sobria* that infects the intestinal tract causes such an extraintestinal infection, the pathogen must pass through the intestinal epithelial barrier. In our earlier study using intestinal cultured cells (T84 cells), we observed that an *A. sobria* strain with higher *A. sobria* serine protease (ASP) production caused a marked level of bacterial translocation across the T84 intestinal epithelial monolayer. Herein, we investigated the effect of ASP on tight junctions (TJs) in T84 cells. We observed that ASP acts on TJs and causes the destruction of ZO-1, ZO-2, ZO-3, and claudin-7 (i.e., some of the protein components constituting TJs), especially in the strains with high ASP productivity. Based on the present results together with those of our earlier study, we propose that ASP may cause a disruption of the barrier function of the intestinal epithelium as a whole due to the destruction of TJs (in addition to the destruction of adherens junctions) and that ASP may assist invasion of the pathogens from the intestinal epithelium into deep sites in the human body.

## Introduction

*Aeromonas sobria* is a Gram-negative bacterium that is widely distributed in both fresh and brackish water areas. *A. sobria* generally causes food-borne illness with watery diarrhea as a main symptom (Janda and Brenden, 1987; Janda and Abbott, 2010), and it opportunistically causes severe extraintestinal diseases including sepsis, peritonitis, and meningitis (Janda and Abbott, 1998; Spadaro et al., 2014; Song et al., 2019; Hutchinson et al., 2021) especially in immunocompromised patients and the elderly (Bhowmick and Bhattacharjee, 2018). These extraintestinal infections can be caused by *A. sobria* that has invaded the body through a wound, but they can be also caused by the bacteria that infect the intestinal tract. If *A. sobria* that infects the intestinal tract causes such an extraintestinal infection, the pathogen must pass through the intestinal epithelial barrier from the initial infection site in the intestinal tract. For the control of these infections, it is thus important to determine how *A. sobria* colonizes in the intestinal tract, invades the intestinal epithelial tissue, and passes through the intestinal barrier.

*A. sobria* extracellularly produces various virulence factors such as cytolysins, enterotoxins, and proteases (Pemberton et al., 1997; Fujii et al., 1998; Okamoto et al., 2000). It is thought that these factors act in concert with each other to increase the pathogenicity of *A. sobria*. We purified a 65-kDa *A. sobria* serine protease (ASP) from the culture supernatant and clarified its crystal structure (Kobayashi et al., 2009). The overall structure of ASP is closely related to that of Kex2 (Leibowitz and Wickner, 1976), a member of the kexin family of subtilases expressed by *Saccharomyces cerevisiae*. We further observed that ASP induces vascular leakage, reduces blood pressure by activating the kallikrein/kinin system (Imamura et al., 2006), promotes human plasma coagulation *via* the activation of prothrombin (Nitta et al., 2007), and causes the formation of pus and edema through the action of anaphylatoxin C5a (Nitta et al., 2008). ASP thus exerts a variety of effects on biological components and is deeply involved in the pathogenicity of *A. sobria*.

Human intestinal epithelial tissue acts as a physical barrier to prevent pathogens that have entered the intestinal tract from advancing out of the intestine. Epithelial cell-to-cell binding is essential to this barrier function. Tight junctions (TJs), adherens junctions (AJs), gap junctions (GJs), and desmosomes contribute as junctional complexes that fulfill cell-cell connections (Farquhar and Palade, 1963). These junctional complexes also interact with the actin cytoskeleton and are involved in controlling the actin cytoskeleton’s function, and they are important for the development of polarized epithelium (Hartsock and Nelson, 2008). The destruction of the above-mentioned intercellular junctions would thus permit the invasion of microbial pathogens, leading directly to the progression of a systemic infection.

TJs, AJs, GJs, and desmosomes are composed of protein components. For example, in TJs, the protein ZO-1 provides a link between the transmembrane proteins (such as occludin and claudin) and the actin cytoskeleton (Fanning et al., 1998). In AJs, the protein afadin provides a link between the transmembrane protein nectin and the actin cytoskeleton, and the protein catenin links the transmembrane protein E-cadherin and the actin cytoskeleton (Hartsock and Nelson, 2008; Takai et al., 2008a; Takai et al., 2008b).

Several virulence factors produced by pathogens were reported to cause the degradation of components related to the cell-cell binding of intestinal cells. Among these factors, HtrA, a serine protease produced by various Gram-negative bacteria, has been the subject of many investigations (Schmidt et al., 2016; Backert et al., 2018; Abfalter et al., 2019; Harrer et al., 2019; Sharafutdinov et al., 2020; Radhakrishnan et al., 2021). We hypothesized that ASP may also contribute to the destruction of these protein components and may evoke bacterial translocation across the intestinal epithelial barrier.

To test this hypothesis, we performed an *in vitro* analysis using cultured T84 cells (Kobayashi et al., 2019), and we observed that ASP acts on AJs and causes the destruction of both nectin-2 and afadin (protein components constituting AJs). We suspect that the destruction of nectin-2 and afadin by the action of ASP (*i*) increases the ability of *A. sobria* to pass through intestinal epithelial tissue, and (*ii*) contributes to the severity of pathological conditions.

However, the intestinal epithelial barrier function is supported not only by AJs but also by other junctional complexes such as TJs, GJs, and desmosomes. In particular, TJs are located on the apical side of the intestinal epithelium, unlike AJs. We thus speculated that ASP may also affect the protein components constituting TJs. To investigate this possibility, we conducted the present study with a more detailed analysis at the molecular level using the same T84 cultured cell line as that used in our earlier investigation (Kobayashi et al., 2019).

## Materials and Methods

### Bacterial Strains and Culture Conditions

Eleven *A. sobria* clinical strains isolated from patients with diarrhea (strains 101, 104, 106, 115, 118, 121, 122, 123, 124, 125, and 288) were used. All strains were genetically confirmed to be *Aeromonas* by a nucleotide sequence analysis using polymerase chain reaction (PCR)-amplified fragments of their 16S rRNA genes (Borrell et al., 1997).

We made a mutant strain in which the *asp* gene of the 288 strain was disrupted as described (Imamura et al., 2008). We designated the mutant strain ‘*A. sobria* 288 Δ*asp*.’ Briefly, disruption of the *asp* gene was carried out by the homologous recombination technique (Kuroda et al., 2005) using a suicide vector, pXAC623 (Nishibuchi et al., 1991; Schweizer and Hoang, 1995). We removed the gene fragment from bp 1,766 to bp 3,408 of the cloned *asp* gene (the deleted gene encodes from the 110th amino acid residue from the amino terminal of ASP to the 26th amino acid residue from the amino terminal of ORF2). We then introduced the derivative plasmid of pXAC623 carrying the mutant *asp* into *E. coli* SM10λ*pir*, from which the plasmid was transferred into wild-type *A. sobria* 288 by conjugation. We selected a mutant strain of *A. sobria* 288 whose wild-type *asp* was replaced with the mutant *asp* by homologous recombination, as described (Herz et al., 2003). Several candidate colonies were selected by PCR using suitable primers, and the mutation of *asp* gene was then confirmed by a Southern blot analysis.

Using the above homologous recombination method, we reintroduced the *asp* gene into the *A. sobria* 288 Δ*asp* strain to create the complementary strain as described (Imamura et al., 2008). We named the newly created strain ‘*A. sobria* 288 Δ*asp*::*asp*.’

Luria-Bertani broth (LB) (BD Biosciences, Franklin Lakes, NJ, USA) and Luria-Bertani agar (BD Biosciences) were used for the shaking culture and the static culture of the *Aeromonas* strains, respectively.

### Purification of ASP

We purified ASP as described (Okamoto et al., 2000). The purity of ASP in the preparation was verified by sodium dodecyl-sulfate polyacrylamide gel electrophoresis (SDS-PAGE) as a sample showing a single band as reported (Fujii et al., 1998; Okamoto et al., 2000).

### Cells and Cell Culture Conditions

The intestinal epithelial cell line T84 was obtained from the European Collection of Authenticated Cell Cultures (ECACC, Porton Down, England). T84 cells were grown and maintained at 37°C in a 5% CO_2_ atmosphere in a 1:1 mixture of Dulbecco’s modified Eagle’s medium (DMEM) and Ham’s F-12 nutrient mixture supplemented with 6% fetal bovine serum (FBS), 15 mM HEPES, 14.3 mM NaHCO_3_, and antibiotics/antimycotics. In the bacterial translocation assay, the cells were grown to a confluent monolayer in collagen-coated polycarbonate Transwell^®^ inserts: 0.33 cm^2^ (Corning Life Sciences, Corning, NY). The culture medium was changed every 3 days. The transepithelial electrical resistance (TER) was measured directly in the culture medium using an epithelial volt–ohm meter (Millicell-ERS^®^; Millipore, Cambridge, MA). The TER values were calculated by subtracting the background TER from blank filters and then multiplying the value obtained by the surface area of the filter. Cells were used for experiments when the TER was between 600 and 1000 ohms•cm^2^, which was 10–14 days post-plating.

In an experiment investigating the degradation of protein components constituting TJs, cells were seeded on a 12-well microplate; the medium was changed every 3 days, and the cells were cultured until they became confluent (culture period 5–7 days).

### Bacterial Translocation Assay

The bacterial translocation assay was carried out as described (Kobayashi et al., 2019). Briefly, bacterial cells obtained from the culture in LB were suspended in Hanks’ balanced salt solution (HBSS) containing 15 mM HEPES (pH 7.4). The suspended bacterial cells (2 x 10^5^ cells) were added at a multiplicity of infection (MOI) of 5 to the apical side of T84 cells grown on Transwell filters (pore size 3.0 µm). In this procedure, the number of bacteria in each bacterial suspension was confirmed by the plating method.

To assess the extent of bacterial translocation from the apical side to the basal side by increasing the number of bacteria present in the lower chamber (the basal side) as much as possible, we performed this experiment for the maximum period (9 hr) during which the T84 intestinal epithelial monolayer did not detach from the Transwell. After infection at 37°C with 5% CO_2_ for 9 hr, the changes in TER were recorded and the number of bacteria present in the culture medium (10 μL) of the lower chamber was calculated by the plating method using LB agar medium (BD Biosciences).

### Measurement of the Proteolytic Activity of ASP and the Immunological Detection of ASP Released Outside the Cell

We measured the proteolytic activity of ASP by using a synthetic peptide, Boc-Glu-Lys-Lys-MCA (Peptide Institute, Osaka, Japan) as described (Kobayashi et al., 2009). The immunological detection of ASP using anti-ASP IgG was carried out as described (Kobayashi et al., 2015).

### Analyses of the Degradation of the Protein Components Constituting TJs by an Immunoblotting Method

We added bacterial cells (MOI = 5) suspended in HBSS containing 15 mM HEPES (pH 7.4) to the apical side of T84 cells cultured in 12-well microplates. In our earlier study, the degradation of the protein components constituting the junctional complexes was observed to start early (Kobayashi et al., 2019). As in that study, we analyzed the degradation of the protein components constituting TJs at 2 hr post-infection. After infection at 37°C with 5% CO_2_ for 2 hr, the monolayer was washed three times in ice-cold phosphate-buffered saline (PBS) and incubated in lysis buffer containing 1% Triton X-100, 100 mM NaCl, 10 mM HEPES, pH 7.5, 2 mM EDTA, and Protease Inhibitor Cocktail (Nacalai Tesque, Kyoto, Japan) for 10 min. Cells were then scraped and passed ten times through a 21-gauge needle. Protein was obtained in the supernatant after centrifugation at 15,000 *g* for 15 min at 4°C. The protein samples were mixed with SDS-sample buffer and heated at 95°C for 5 min before loading onto 15% SDS-polyacrylamide gels.

For the evaluation of the effect of protease inhibitors on the degradation of the protein components constituting TJs, we added bacterial cells of *A. sobria* strain 288 suspended in HBSS containing 15 mM HEPES and pH 7.4 (MOI = 5) to the apical side of T84 cells cultured in 12-well microplates in the presence of various types of protease inhibitors. The concentrations of the protease inhibitors used were as follows: 15 mM pepstatin A (P5318, Sigma-Aldrich, St. Louis, MO), 5.5 mM chymostatin (C7268, Sigma-Aldrich), 21 mM leupeptin (L2884, Sigma-Aldrich), 1 mM phenylmethylsulfonyl fluoride (PMSF; 06297-31, Nacalai Tesque), and 1 mM ethylenediaminetetraacetic acid (EDTA; 15130-95, Nacalai Tesque). After these treatments, the electrophoresis samples were prepared by the method described above.

After electrophoresis the proteins were transferred to PVDF membranes (pore size 0.2 µm, Bio-Rad, Hercules, CA). We detected proteins by immunoblotting using primary antibodies against ZO-1 (610966, BD Biosciences) 1:1000 (dilution ratio); ZO-2 (71-1400, Thermo Fisher Scientific, Waltham, MA) 1:250; TJP3 (ZO-3) (HPA053337, Sigma-Aldrich) 1:1000; occludin (33-1500, Thermo Fisher Scientific) 1:500; claudin-1 (37–4900, Thermo Fisher Scientific) 1:250; claudin-3 (ab15102, Abcam, Cambridge, UK) 1:500; claudin-4 (329400, Thermo Fisher Scientific) 1:500; claudin-7 (ab27487, Abcam) 1:100; and GAPDH (016–25523, Wako Pure Chemical Industries, Osaka, Japan) 1:1000. The secondary antibody was horseradish peroxidase (HRP)-conjugated goat anti-rabbit IgG (111-035-003, Jackson Immunoresearch Laboratories, West Grove, PA) 1:10000 (dilution ratio) or HRP-conjugated goat anti-mouse IgG (115-035-003, Jackson Immunoresearch Laboratories) 1:10000 (dilution ratio). A Clarity™ Western ECL substrate (Bio-Rad) was used to detect the positive bands. All experiments were performed in triplicate.

### Immunofluorescence Confocal Laser Scanning Microscopy

T84 cells were grown on Transwell filters (pore size 0.4 µm) and treated with various bacterial solutions suspended in HBSS containing 15 mM HEPES and pH 7.4 at MOI = 5. After incubation for 3 hr at 37°C in a 5% CO_2_ atmosphere, the monolayer was washed three times in ice-cold PBS and fixed/permeabilized in ice-cold 100% ethanol at −20°C for 20 min. After being blocked with BlockAid™ Blocking Solution (Thermo Fisher Scientific), the samples were incubated with the primary antibodies against ZO-1 (610966, BD Biosciences) 1:500 and claudin-7 (ab27487, Abcam) 1:100. The secondary antibody was Cy5-conjugated goat anti-rabbit antibody (ab6564, Abcam) 1:1000 or Alexa Fluor 594-conjugated goat anti-mouse antibody (ab150116, Abcam) 1:1000. Samples were washed with PBS, and then 4’,6-diamidino-2-phenylindole (DAPI; Thermo Fisher Scientific) was applied for 10 min to stain the nuclei. After a final wash with PBS, the membranes were transferred to a glass slide and mounted in ProLong™ Gold mount gel (Thermo Fisher Scientific). Fluorescence signals were visualized with a confocal laser scanning microscope (LSM800, Carl Zeiss, Oberkochen, Germany).

### Statistical Analyses

Experimental data were analyzed using GraphPad Prism software (La Jolla, CA). Comparisons between two datasets were performed using the unpaired Student’s *t*-test. We also tested the significance of differences in some of the data by a one-way analysis of variance (ANOVA) using EZR and the interface program R (Dunnett’s test).

## Results

### Changes in Barrier Function due to the Addition of Various *A. sobria* Strains to the T84 Intestinal Epithelial Monolayer

**Figure 1:**
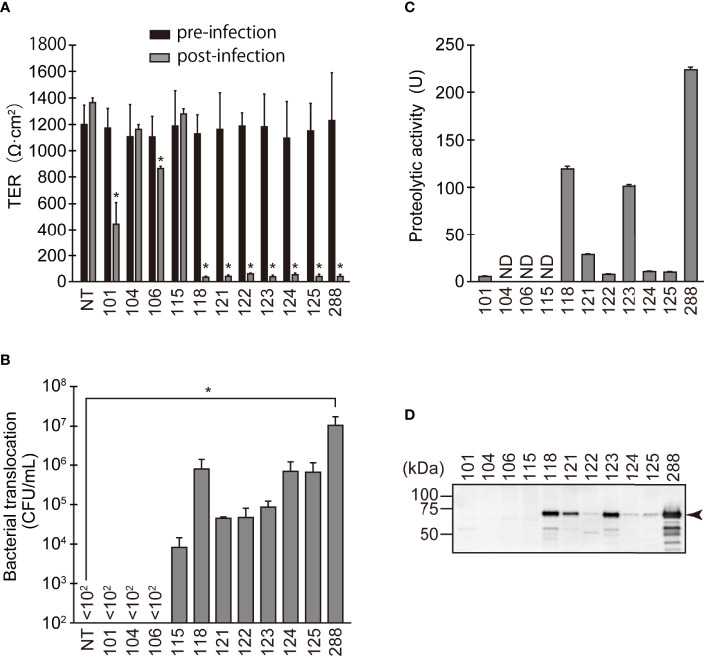
Changes in barrier function due to infection of various *A. sobria* strains to the apical side of the T84 intestinal epithelial monolayer. **(A)** T84 cells were grown on a Transwell system and then infected with several *A. sobria* strains. After 9 hr of infection (MOI = 5) to the apical side of the T84 monolayer, the TER was measured (post-infection). The obtained TER value was compared with the TER value before infection (pre-infection). NT: The TER value was measured without bacterial infection. The experiments were performed in triplicate. The data are mean ± SD (error bars). We performed an ANOVA analysis with Dunnett’s test and showed significant differences (*p < 0.05) between the data obtained from each bacterial strain and the control data (NT). **(B)** The ability of the *A. sobria* strains to translocate across the T84 intestinal epithelial monolayer at 9 hr after infection (MOI = 5) was evaluated by calculating the colony forming units per mL (CFU/mL) of the culture medium in the lower chambers (the basal side of the T84 monolayer). The number of bacteria present in the solution (10 μL) of the lower chamber was calculated by the plating method. In this method, the number of bacteria less than 100 CFU/mL cannot be determined. If no bacteria were found in 10 μL of solution in the lower chamber, it was displayed as “<10^2^” in the figure. The experiments were performed in triplicate. The data are mean ± SD (error bars). We performed an ANOVA analysis with Dunnett’s test and showed significant differences (*p < 0.05) between the data obtained from each bacterial strain and the control data (NT). **(C)** The proteolytic activity in the culture supernatant of each *A. sobria* strain was measured as described in the text. The experiments were performed in triplicate. ND: The proteolytic activity could not be sufficiently detected in this experimental condition. The data are mean ± SD (error bars). **(D)** The presence of ASP in the culture supernatant of each strain was immunologically detected by a western blotting analysis as described in the text. The *arrow* indicates the position of the ASP (65-kDa) band fractionated by SDS-PAGE.

We first examined changes in barrier function due to the addition of the eleven *A. sobria* strains to the T84 intestinal epithelial monolayer. We measured the electrical resistance (TER) of the T84 intestinal epithelial monolayer before (pre-infection) and after (post-infection) treatment with various *A. sobria* strains (**Figure 1A**). To assess the extent of bacterial translocation from the apical side to the basal side of the T84 monolayer, we counted the number of bacteria present in the lower chamber (the basal side) (**Figure 1B**). We also measured the ASP activity in the culture supernatant of each strain (**Figure 1C**), and the existence of ASP in the culture supernatant was determined by an immunoblot analysis using anti-ASP antibody (**Figure 1D**). These experimental procedures were carried out as described (Kobayashi et al., 2019).

The three strains with the higher ASP productivity (strains 118, 123, and 288) caused a significant decrease in the TER value. Four strains (121, 122, 124, and 125) also caused a significant decrease in the TER value, although their ASP productivity was lower than that of the above three strains. In agreement with these results, bacterial translocation across the T84 monolayer was clearly seen in these seven strains. However, only the data obtained from strain 288, which showed the highest ASP productivity, showed a significant difference from the control data (NT).

In contrast, strain 101, which has low ASP productivity, did not cause bacterial translocation across the T84 monolayer, even though the strain caused a decrease in the TER value to some extent with a significant difference. In addition, two strains without ASP productivity (104 and 106) did not cause bacterial translocation across the T84 monolayer although strain 106 caused a slight decrease in the TER value with a significant difference. Surprisingly, strain 115 allowed bacterial translocation across the T84 monolayer even though it lacks ASP activity and did not alter the TER.

### Degradation of Protein Components of TJs Caused by the Addition of *Aeromonas* Strains to the T84 Intestinal Epithelial Monolayer

**Figure 2:**
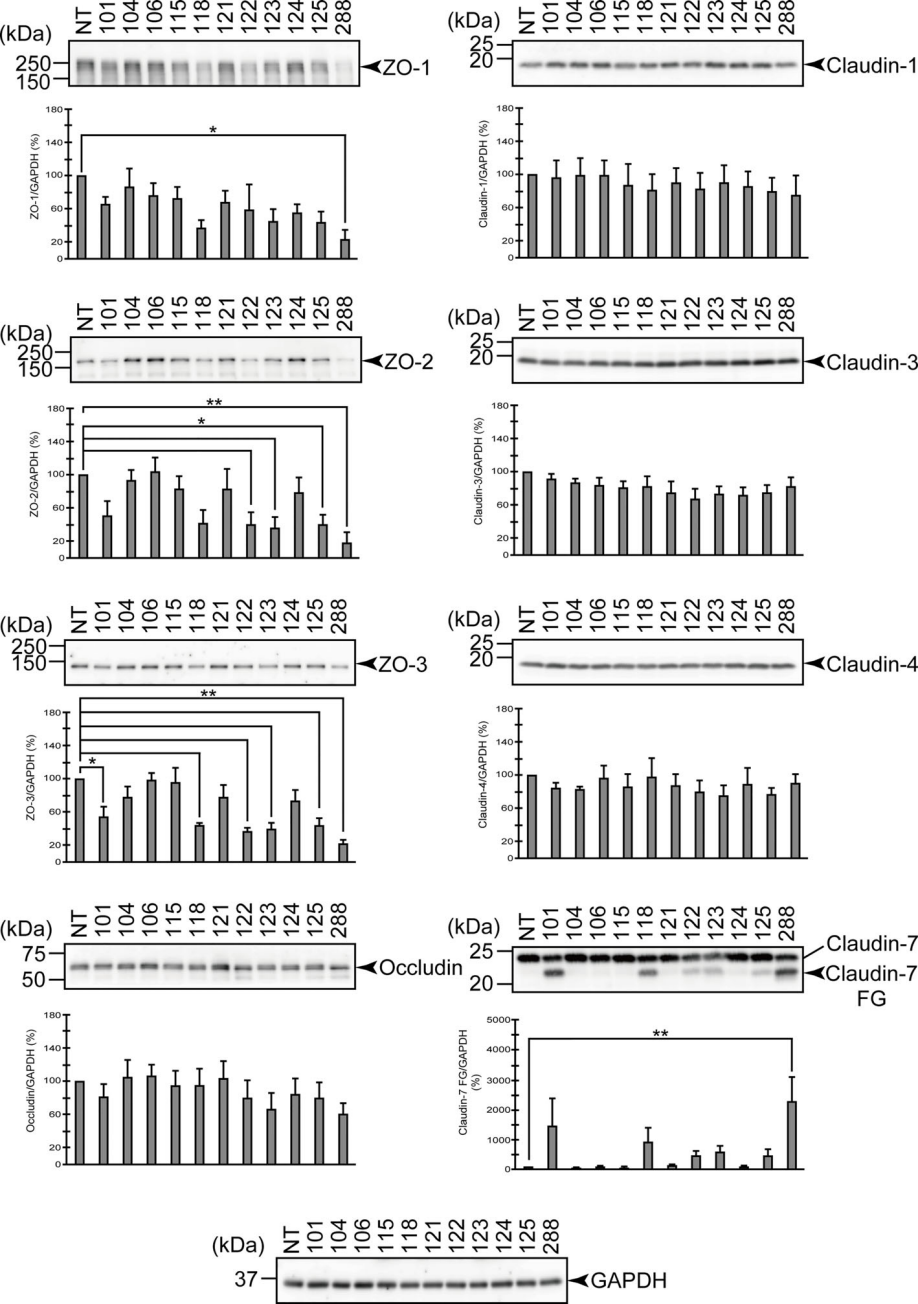
Degradation of the protein components constituting TJs due to infection of various *A. sobria* strains to the apical side of the T84 intestinal epithelial monolayer. T84 cells were cultured in 12-well microplates and then infected with several *A. sobria* strains. After 2 hr of infection (MOI = 5) to the apical side of the T84 monolayer, the cell extracts were prepared. The protein components constituting TJs (ZO-1, ZO-2, ZO-3, occludin, and claudin-1, -3, -4, and -7) were detected by using a specific antibody against each protein. The results of the quantitative analysis of the relative ratio (%) of the amount of blotted protein (ZO-1, ZO-2, and ZO-3) to the amount of GAPDH are shown below the western blotting image. Concerning the degradation of claudin-7, the results of the quantitative analysis of the relative ratio (%) of the amount of the blotted claudin-7 degradation fragment (Claudin-7 FG) to the amount of GAPDH are shown. These experiments were performed in triplicate. The data are mean ± SD (error bars). We performed an ANOVA analysis with Dunnett’s test and showed significant differences (*p < 0.05, **p < 0.01) between the data obtained from each bacterial strain and the control data (NT).

We next examined the effects on the protein components of TJs using the T84 culture cells in greater detail. **Figure 2** illustrates the degradation of various protein components constituting TJs 2 hr after the addition of the *A. sobria* strains to the apical side of the T84 intestinal epithelial monolayer. The degradation of the protein components was quantitatively analyzed in a comparison of the amounts of GAPDH, which is constitutively expressed intracellularly, as a control.

From the results shown in **Figure 2**, the three above-mentioned strains (strains 118, 123, and 288) that showed remarkable ASP productivity seemed to cause the degradation of ZO-1, ZO-2, ZO-3, and claudin-7. Among these strains, strain 288 with the highest ASP productivity caused significant degradation of both the ZO proteins and claudin-7. However, the two strains other than strain 288 did not always show a significant difference in the degradation of these proteins. On the other hand, none of these three strains degraded occludin, claudin-1, -3, or -4 with a significant difference. In contrast, the three strains without ASP productivity (104, 106, and 115) did not significantly degrade any of the ZO proteins or claudin.

For the other strains that showed low ASP productivity (101, 121, 122, 124, and 125), the results were complicated, except that none of these strains degraded occludin, claudin-1, -3, or -4 significantly.

The findings for four strains (121, 122, 124, and 125) that caused both a marked decrease in the TER value and bacterial translocation across the T84 monolayer (**Figure 1**) were as follows. Judging from the results shown by Western blotting, strains 122 and 125 seem to cause degradation of ZO-1, ZO-2, ZO-3, and claudin-7 to some extent. However, only the degradation of ZO-2 and ZO-3 showed a significant difference at the time of observation. In contrast, strains 121 and 124 did not significantly degrade either the ZO proteins or claudin-7.

On the other hand, strain 101 seems to cause degradation of ZO-1, ZO-2, ZO-3, and claudin-7 to some extent as seen in strains 122 and 125, but only the degradation of ZO-3 showed a significant difference at the time of observation.

### The Effects of the Protease Inhibitors on the Degradation of the Protein Components Constituting TJs

The results shown in **Figure 2** seem to imply that it is necessary to consider the possibility of various effects in strains with weak ASP productivity (these will be mentioned in the Discussion section). However, we observed that the strains with marked ASP productivity caused the significant degradation of both ZO proteins and claudin-7 and induced a decrease in the barrier function of the T84 intestinal epithelial monolayer. Therefore, we next focused on the contribution of ASP to the degradation of ZO proteins and claudin-7 by using strain 288, which showed the highest ASP productivity.

**Figure 3:**
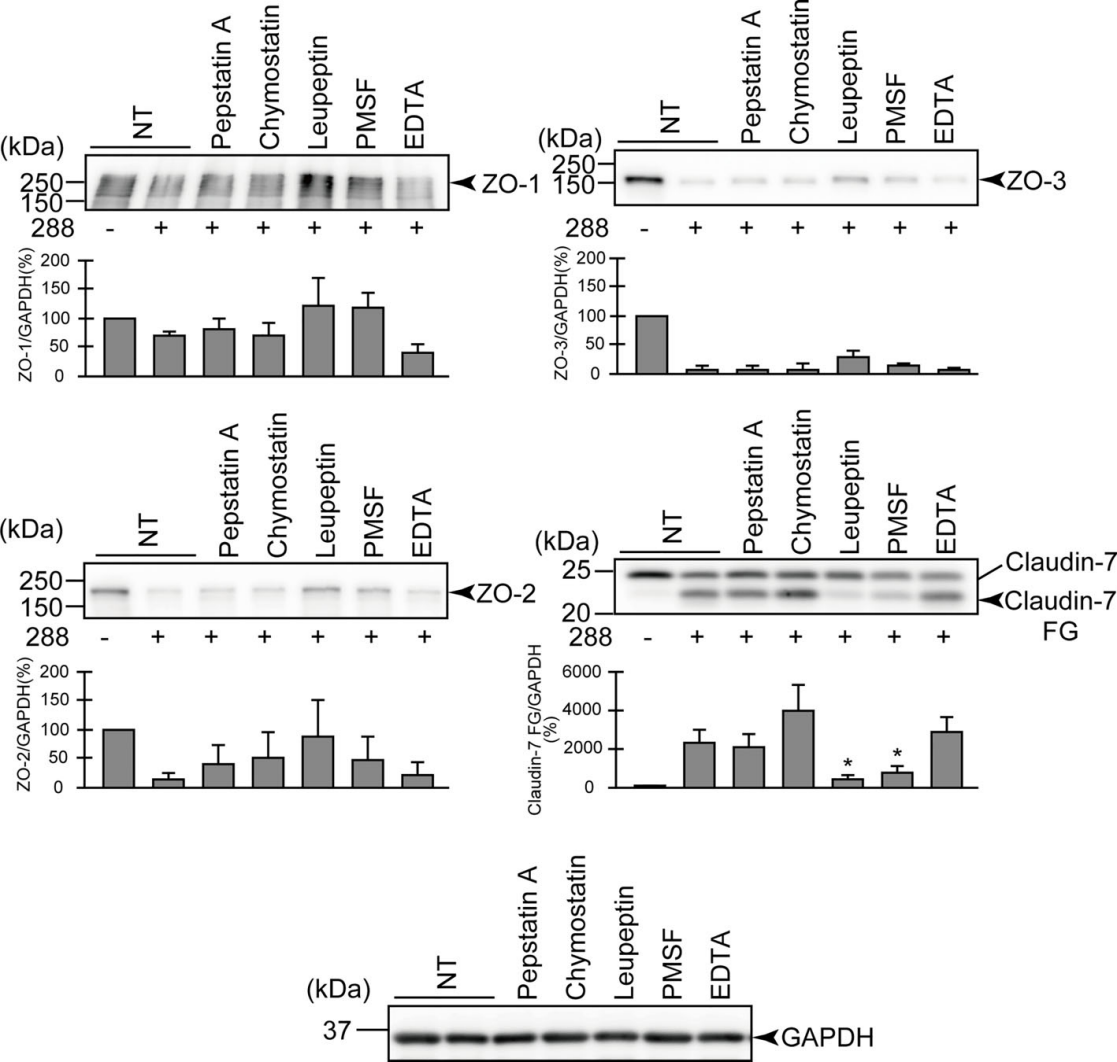
Effects of various protease inhibitors on the degradation of ZO-1, ZO-2, ZO-3, and claudin-7 due to infection of *A. sobria* strain 288 to the apical side of the T84 intestinal epithelial monolayer. T84 cells were cultured in 12-well microplates and then infected with *A. sobria* strain 288 in the presence of the protease inhibitors shown. After 2 hr of infection (MOI = 5) to the apical side of the T84 monolayer, the cell extracts were prepared. The protein components (ZO-1, ZO-2, ZO-3, and claudin-7) constituting TJs were detected by using a specific antibody against each protein. The results of the quantitative analysis of the relative ratio (%) of the amount of blotted protein (ZO-1, ZO-2, and ZO-3) to the amount of GAPDH are shown below the western blotting image. Concerning the degradation of claudin-7, the results of the quantitative analysis of the relative ratio (%) of the amount of the blotted Claudin-7 FG to the amount of GAPDH is shown. These experiments were performed in triplicate. The data are mean ± SD (error bars). *p < 0.05; significance was tested by comparison with the control data (NT; without infection with strain 288).

We first examined the effect of several protease inhibitors on the infection of the apical side of the T84 monolayer. We used several protease inhibitors: pepstatin A (an acidic protease inhibitor), chymostatin (a chymotrypsin inhibitor), leupeptin (a peptidic protease inhibitor for cysteine, serine, or threonine proteases), PMSF (a low-molecular-weight serine protease inhibitor), and EDTA (a metalloprotease inhibitor). As shown in **Figure 3**, the degradation of ZO-1, ZO-2, and ZO-3 tended to be suppressed by the addition of leupeptin or PMSF compared to the addition of the other inhibitors. The generation of a degraded fragment of claudin-7 (Claudin-7 FG; **Figure 3**) associated with the degradation of claudin-7 was significantly inhibited by leupeptin and by PMSF. It is therefore likely that ASP, the serine protease produced by *A. sobria*, contributes to the degradation of ZO-1, ZO-2, ZO-3, and claudin-7 in the strains with sufficient ASP productivity.

### The Effect of the *asp* Gene Disruption on the Degradation of the Protein Components Constituting TJs

**Figure 4:**
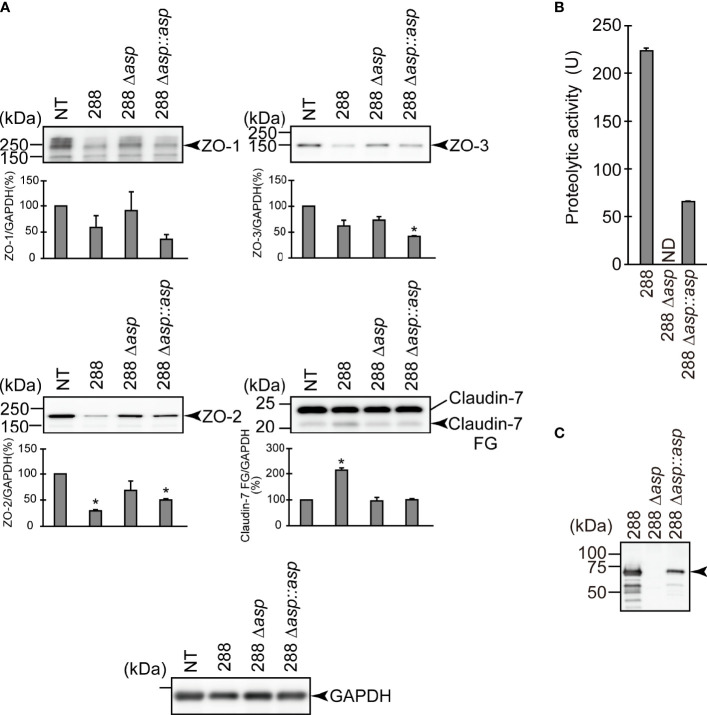
Effect of the *asp* gene disruption on the degradation of the protein components constituting TJs. **(A)** T84 cells were cultured in 12-well microplates and then infected with *A. sobria* strain 288 and its derivative strains shown in the figure. After 2 hr of infection (MOI = 5) to the apical side of the T84 monolayer, the cell extracts were prepared. The protein components (ZO-1, ZO-2, ZO-3, and claudin-7) constituting TJs were detected by using a specific antibody against each protein. The results of the quantitative analysis of the relative ratio (%) of the amount of blotted protein (ZO-1, ZO-2, and ZO-3) to the amount of GAPDH are shown below the western blotting image. Concerning the degradation of claudin-7, the results of the quantitative analysis of the relative ratio (%) of the amount of the blotted Claudin-7 FG to the amount of GAPDH are shown. These experiments were performed in triplicate. The data are mean ± SD (error bars). *p < 0.01; significance was tested by comparison with the control data (NT). **(B)** The proteolytic activity in the culture supernatant of each *A. sobria* strain was measured as described in the text. The experiments were performed in triplicate. ND: The proteolytic activity could not be sufficiently detected in this experimental condition. The data are mean ± SD (error bars). **(C)** The presence of ASP in the culture supernatant of each strain was immunologically detected by a western blotting analysis as described in the text. The *arrow* indicates the position of the ASP (65-kDa) band fractionated by SDS-PAGE.

To further evaluate the contribution of ASP to the degradation of the protein components constituting TJs, we carried out a similar *in vitro* infection study using an *A. sobria* mutant in which the *asp* gene was knocked out (288 Δ*asp*). As shown in **Figure 4**, the degradation of ZO-1, ZO-2, ZO-3, and claudin-7 was decreased by the addition of the 288 Δ*asp* mutant to the T84 intestinal epithelial monolayer. In contrast, the reduction of the degradations of ZO-1, ZO-2, and ZO-3 was restored to some extent when we used the strain complemented with the knocked-out gene (**Figure 4**, 288 Δ*asp*::*asp*). Although the results of the *in vitro* study also confirmed that the complementary strain secreted ASP outside the cells (**Figures 4B, C**), the degradation of claudin-7 by the complementary strain was weak and did not show a significant difference.

### The Contribution of ASP to the Degradation of the Protein Components Constituting TJs

**Figure 5:**
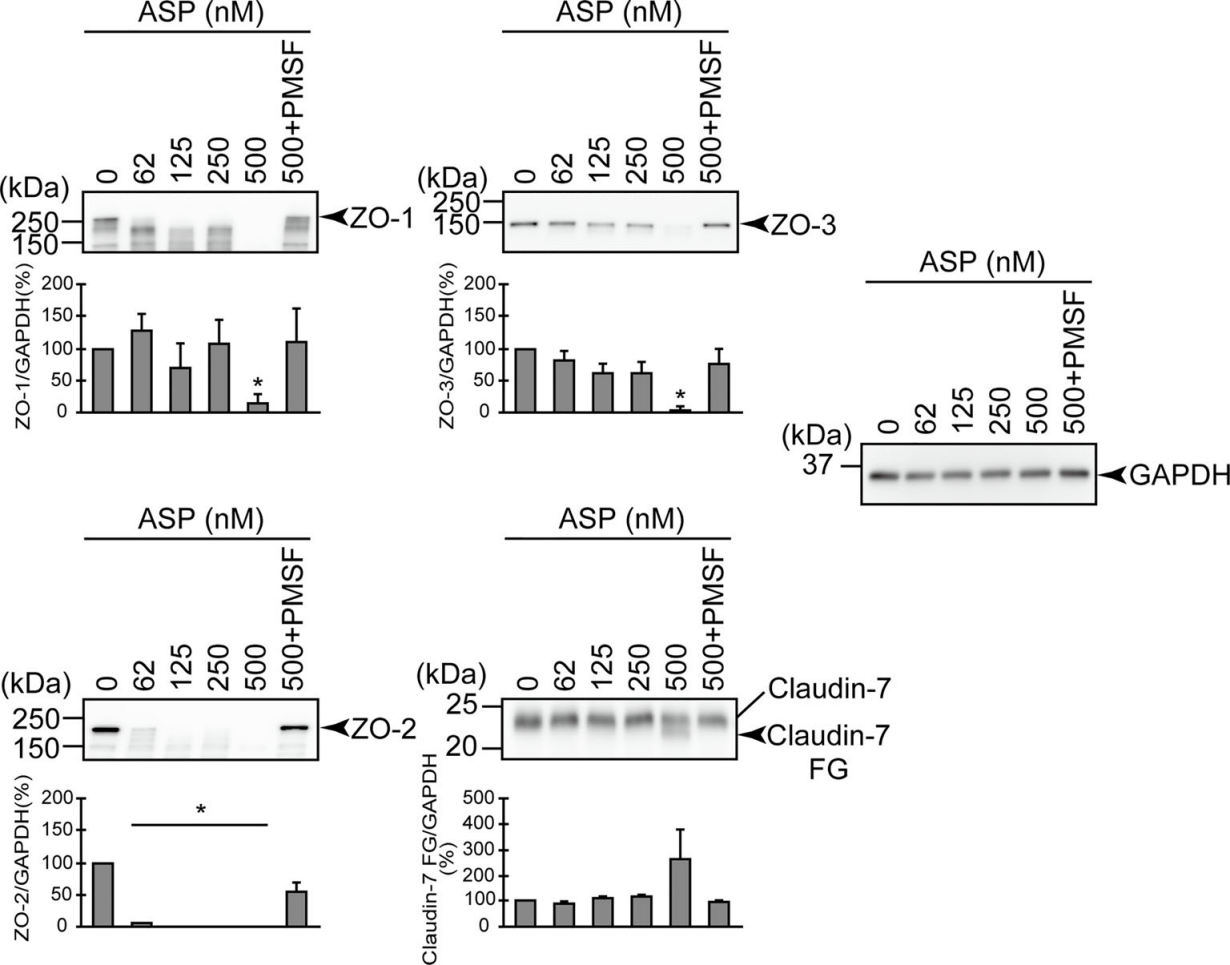
Effect of the purified ASP on various protein components constituting TJs. T84 cells were cultured in 12-well microplates and then treated with various concentrations (nM) of the purified ASP. For the evaluation of the effect of the serine protease inhibitor, T84 cells were also treated with 500 nM of the purified ASP in the presence of PMSF. After treatment for 2 hr, the cell extracts were prepared. The protein components (ZO-1, ZO-2, ZO-3, and claudin-7) constituting TJs were detected by using a specific antibody against each protein. The results of the quantitative analysis of the relative ratio (%) of the amount of blotted protein (ZO-1, ZO-2, and ZO-3) to the amount of GAPDH are shown below the western blotting image. Regarding the degradation of claudin-7, the results of the quantitative analysis of the relative ratio (%) of the amount of the blotted Claudin-7 FG to the amount of GAPDH are shown. These experiments were performed in triplicate. The data are mean ± SD (error bars). *p < 0.01; significance was tested by comparison with the control data (0 nM ASP).

To clarify whether ASP itself engages in the degradation of ZO-1, ZO-2, ZO-3, and claudin-7, we directly added purified ASP to the T84 intestinal epithelial monolayer. The results revealed that ASP is involved in the degradation of ZO-1, ZO-2, and ZO-3 in a dose-dependent manner. In contrast, the degradation of claudin-7 by ASP was weak, even with a high concentration (500 nM) of ASP (**Figure 5**).

### The Contributions of Factors Other Than ASP to the Degradation of the Protein Components of TJs

**Figure 6:**
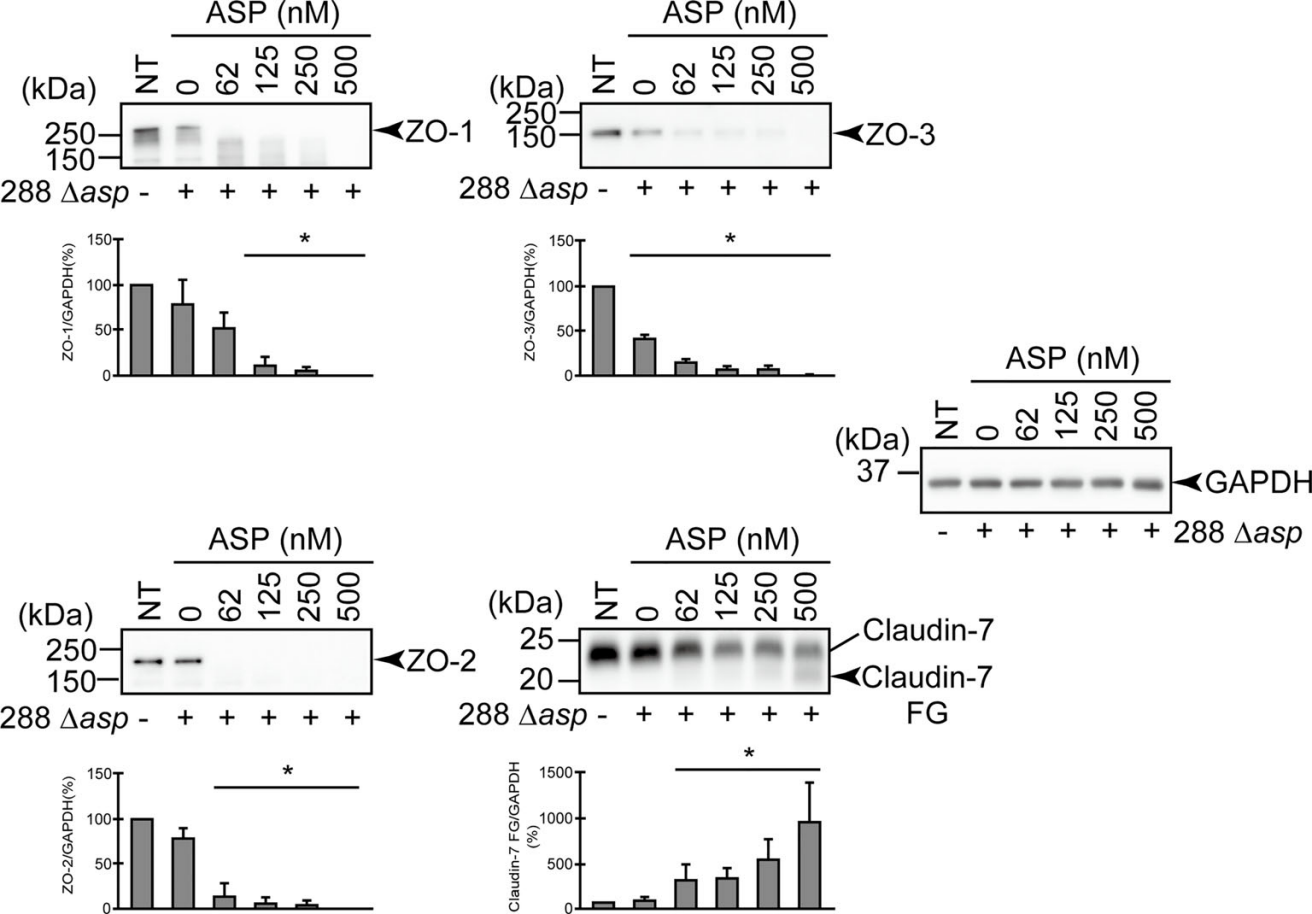
Degradation of ZO-1, ZO-2, ZO-3, and claudin-7 due to infection of the *asp*-deficient *A. sobria* strain (288 Δ*asp*) to the apical side of the T84 intestinal epithelial monolayer in the presence of various concentrations of the purified ASP. T84 cells were cultured in 12-well microplates and then infected with the *A. sobria* 288 Δ*asp* strain in the presence of various concentrations of the purified ASP. After 2-hr treatment, the cell extracts were prepared. ZO-1, ZO-2, ZO-3, and claudin-7 were detected by using a specific antibody against each protein. The results of the quantitative analysis of the relative ratio (%) of the amount of blotted protein (ZO-1, ZO-2, and ZO-3) to the amount of GAPDH are shown below the western blotting image. Concerning the degradation of claudin-7, the results of the quantitative analysis of the relative ratio (%) of the amount of the blotted Claudin-7 FG to the amount of GAPDH are shown. These experiments were performed in triplicate. The data are mean ± SD (error bars). *p < 0.01; significance was tested by comparison with the control data (NT).

The results depicted in **Figure 5** indicated that one or more factors other than ASP may be involved in the degradation of protein components of TJs, especially that of claudin-7. To examine this possibility, we performed an *in vitro* infection study using the *A. sobria* mutant in which the *asp* gene was knocked out (288 Δ*asp*) in the presence of various concentrations of purified ASP. The results are depicted in **Figure 6**. Unlike the results in **Figure 5**, the degradation of ZO-1, ZO-2, ZO-3 and even claudin-7 progressed efficiently in this experimental condition. These results indicate that one or more factors other than ASP produced by the *asp*-knockout strain may be involved in the efficient degradation of these protein components of TJs in cooperation with ASP, although the identity of the factor(s) is uncertain at present.

### CLSM Observation of the Degradation of the TJs’ Protein Components

**Figure 7:**
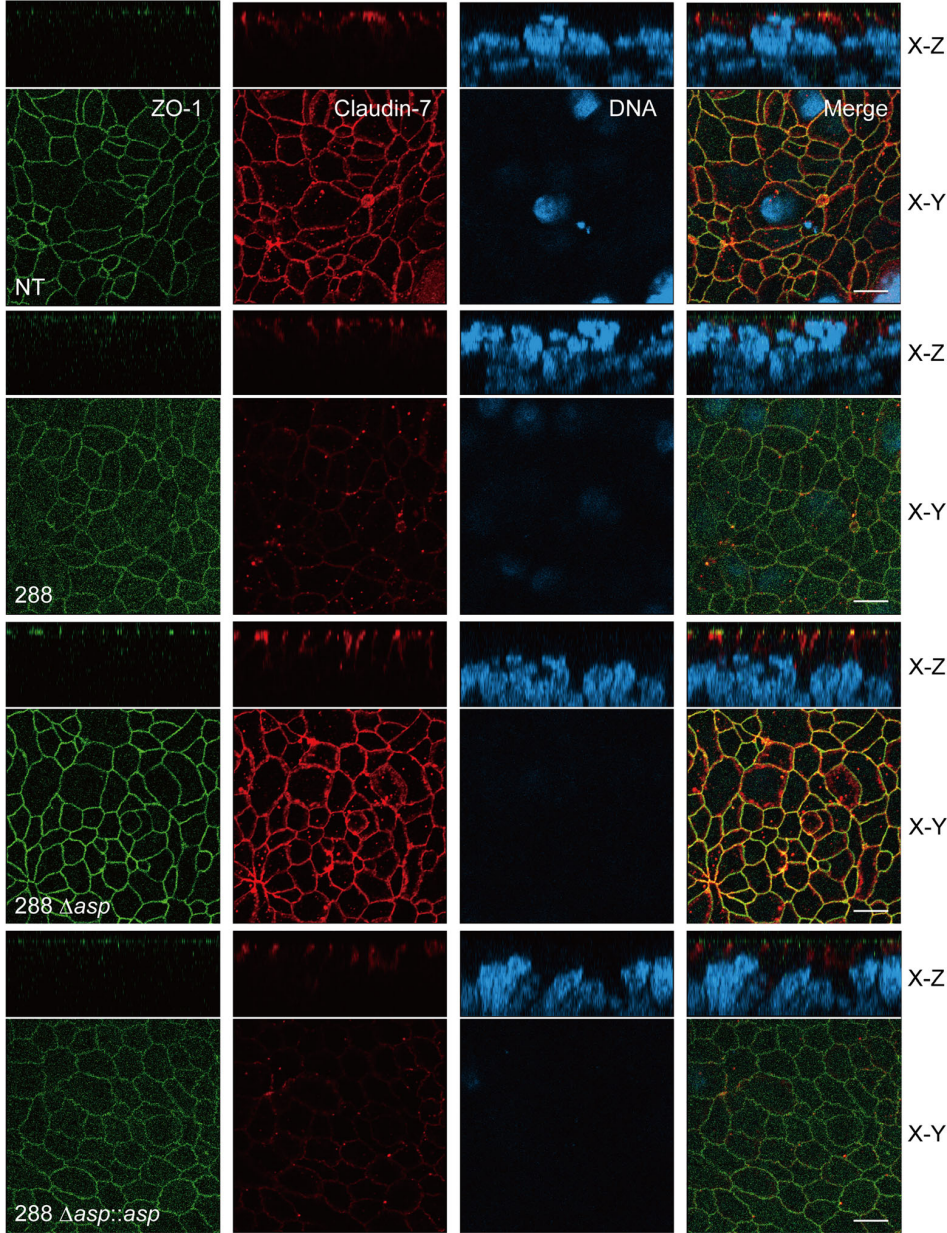
Confocal images of the T84 intestinal epithelial monolayer infected with *A. sobria* strain 288 and its derivative strains. T84 cells were grown on a Transwell system and then infected with *A. sobria* strain 288 and its derivative strains, *A. sobria* strain 288 Δ*asp* and *A. sobria* strain 288 Δ*asp*::*asp*. ZO-1 (*green fluorescence*) and claudin-7 (*red fluorescence*) were immunologically observed. Intracellular DNA was also immunologically detected (*blue fluorescence*). x-y: The x-y image of the T84 intestinal epithelial monolayer surface. x-z: Cross-sectional image of the deeper layer. *White bars:* 10 μm.

The above-described results indicate that ASP is involved in the degradation of the protein components constituting TJs, although one or more additional factors may be indispensable for the degradation of claudin-7. To examine the degradation of the protein components visually, we used confocal laser scanning microscopy (CLSM). We focused on the degradation of ZO-1 and claudin-7 of the T84 intestinal epithelial monolayer by using three strains: *A. sobria* strain 288 (the wild-type strain), the mutant *A. sobria* strain 288 Δ*asp*, and its complemented strain, *A. sobria* strain 288 Δ*asp*::*asp*. The protein components ZO-1 and claudin-7 were visualized using anti-ZO-1 and anti-claudin-7 antibodies and the fluorescence-labeled secondary antibody. As expected, both ZO-1 and claudin-7 were localized in the intercellular adhesion part of the cell surface in the control experiment (**Figure 7**, NT, the x-y image).

Compared to the results of the control experiment, the fluorescence intensities of ZO-1 and claudin-7 were markedly diminished in the T84 intestinal epithelial monolayer treated with *A. sobria* strain 288, which produces a high amount of ASP (**Figure 7**, 288). This phenomenon was not observed in the same experiment using the *A. sobria* mutant in which the *asp* gene was knocked out (**Figure 7**, 288 Δ*asp*). In addition, a decrease in the fluorescence intensities of ZO-1 and claudin-7 was caused in the same experiment when the complemented strain, *A. sobria* strain 288 Δ*asp*::*asp* was used (**Figure 7**).

## Discussion

*A. sobria* opportunistically causes severe extraintestinal diseases, especially in immunocompromised patients and elderly individuals (Spadaro et al., 2014; Bhowmick and Bhattacharjee, 2018; Hutchinson et al., 2021). Such extraintestinal infectious diseases have resulted in fatalities in Japan (Shimoji et al., 2015; Nakao et al., 2016). As noted in the Introduction, this pathogenic bacterium must pass through the intestinal epithelial barrier of the intestinal tract in order to translocate from the intestinal infection site to outside of the intestinal tract, thus causing an extraintestinal infection.

Various pathogenic microorganisms are known to target intercellular adhesion factors (Zihni et al., 2014; Drolia and Bhunia, 2019; Huber, 2020). *Campylobacter jejuni* HtrA cleaves the TJ components claudin-8 (Sharafutdinov et al., 2020) and occludin (Harrer et al., 2019), and *Helicobacter pylori* HtrA recognizes the signature motif of E-cadherin and causes cleavage (Schmidt et al., 2016). It is thus established that the action of the bacterial serine protease HtrA produced by various Gram-negative bacteria is deeply involved in the degradation of protein components that constitute junctional complexes.

In the present study, we infected several *Aeromonas* strains on the apical side of the T84 intestinal epithelial monolayer and investigated their effects on TJs. Based on the results depicted in **Figures 1** and **2**, it is apparent that the characteristics of the *Aeromonas* strains used herein should be discussed separately.

The *Aeromonas* strains showing high ASP productivity (strains 118, 123, and 288) exhibit the following characteristics. They caused a marked decrease in the TER value of the T84 monolayer, and the bacterial translocation across the T84 monolayer progressed in concert with the decrease in the TER value. Since all three of these strains (especially strain 288) caused degradations of ZO-1, ZO-2, ZO-3, and claudin-7 after infection with the T84 monolayer, we suspect that ASP produced outside the cells may cause or contribute to (*i*) the degradation of these protein components and (*ii*) a decrease in the barrier function of the T84 intestinal epithelial cells. It is likely that these actions assist bacterial translocation across the T84 monolayer. Thus, in *Aeromonas* strains that produce a high amount of ASP, the effect of ASP on TJs appears to be closely related to tissue invasiveness. We thus further investigated the contribution of ASP to the degradation of ZO proteins and claudin-7 by using the strain 288, which showed the highest ASP productivity.

The experiment using several protease inhibitors revealed that (*i*) the degradations of ZO-1, ZO-2, and ZO-3 tended to be suppressed by the addition of leupeptin or PMSF, and (*ii*) the generation of a degraded fragment of claudin-7 associated with the degradation of claudin-7 was significantly inhibited by leupeptin and by PMSF (**Figure 3**). Since both leupeptin and PMSF inhibit serine protease, we speculate that these inhibitors attenuated the action of ASP and suppressed the degradation of ZO proteins and claudin-7.

To further clarify the contribution of ASP to the degradation of ZO proteins and claudin-7, we conducted an *in vitro* infection experiment using the *A. sobria* 288 mutant strain in which the *asp* gene was knocked out (288 Δ*asp*). Since a degradation of ZO-1, ZO-2, ZO-3, and claudin-7 was not observed when the T84 intestinal epithelial monolayer was infected with the mutant strain 288 Δ*asp*, it appears likely that ASP produced by the *A. sobria* strain contributed to the degradation of those TJ protein components (**Figure 4**). The experiment using the strain complemented with knocked-out gene (288 Δ*asp*::*asp*) revealed that the degradation of ZO-1, ZO-2, and ZO-3 occurred again to some extent but the degradation of claudin-7 did not occur to a similar degree. Although the reason for the inadequate recovery of degradation by the complementary strain is unknown, it is possible that the amount of ASP produced in the complementary strain was lower than that in the parent strain (**Figures 4B, C**). A different approach in a future study may clarify this result.

We then added purified ASP to the T84 intestinal epithelial monolayer to directly determine whether ASP is involved in the degradation of TJ protein components. The results demonstrated that the addition of purified ASP to the T84 intestinal epithelial monolayer resulted in the degradation of ZO-1, ZO-2, and ZO-3 in a dose-dependent manner (**Figure 5**). However, the degradation of claudin-7 by purified ASP was weak, even with 500 nM ASP, which is much higher than the ASP concentration detected in the culture supernatant (**Figure 5**). We thus speculate that one or more factors other than ASP may also cause the degradation of TJ protein components, especially claudin-7, while there is no doubt that ASP is involved in the degradation of claudin-7.

To investigate whether unknown factor(s) other than ASP are involved in the degradation of claudin-7, we carried out an *in vitro* infection study using the *A. sobria* mutant (288 Δ*asp*) in the presence of various concentrations of purified ASP. As shown in **Figure 6**, the degraded fragment of claudin-7 was confirmed when the *asp* gene-knocked-out strain was infected in the T84 intestinal epithelial monolayer in the presence of purified ASP compared to the case in which purified ASP alone was applied to the monolayer. This result suggests that unknown factor(s) other than ASP produced by the *A. sobria* strain cooperate with ASP to cause the degradation of claudin-7, although the identity of the factor(s) is uncertain at present. These findings are of great interest, and we are planning further research to elucidate the underlying molecular mechanisms, including the identification of the unknown factor(s).

We used CLSM to visually confirm the degradation of the protein components constituting TJs after the T84 intestinal epithelial monolayer was treated with the *A. sobria* strain 288 derivatives. The results shown in **Figure 7** revealed that marked degradations of ZO-1 and claudin-7 occurred when the T84 intestinal epithelial monolayer was infected with the ASP-producing *A. sobria* strains (i.e., the wild-type 288 strain and the complementary strain 288 Δ*asp*::*asp*), but these degradations did not occur when the monolayer was treated with the *A. sobria* mutant strain in which the *asp* gene was knocked out. We therefore believe that ASP produced from the *A. sobria* strain acts as at least a major factor in the degradation of protein components constituting TJs such as ZO-1 and claudin-7.

The present findings thus indicate that ASP causes a degradation of protein components that constitute TJs, at least in the *Aeromonas* strains with higher ASP productivity. There are some other aspects to consider, however. For example, ZO proteins are known to be intracellular scaffold proteins that function in concert with transmembrane proteins such as claudins that are localized on the cell surface (Steed et al., 2010). ASP released from the *A. sobria* strains to the outside of the cells may act directly on the protein localized on the cell surface but cannot directly act on the proteins existing inside the cells. The question thus arises: how can ASP act on intracellularly localized scaffold proteins such as ZO-1, ZO-2, and ZO-3?

The characteristic structure of TJs is thought to be formed primarily by the accumulation of claudin molecules (Rosenthal et al., 2012). The ZO proteins function by directly binding to transmembrane proteins such as claudin and intracellular actin filaments, and this promotes the accumulation of the membrane proteins to form complete TJs (Itoh et al., 1997; Umeda et al., 2006). The maintenance of epithelial TJs has been shown to be functionally regulated by ZO molecules and the Rho signaling pathway (Itoh et al., 2012). Thus, the protein components constituting TJs are closely linked to each other and contribute to the maintenance of the epithelial barrier function. The barrier function is therefore thought to be significantly impaired no matter where any of these protein factors break down.

When a bacterial virulence factor such as ASP acts on a specific protein component of TJs, it is likely that the homeostasis of the entire TJ will be lost and other protein components of TJs will be also destroyed accordingly. Since ASP is extracellularly secreted from the bacterial cells, we suspect that the target molecule on which ASP acts first is a cell-surface component such as the claudins. Accordingly, we suspect that intracellular scaffold proteins such as ZO proteins may also be damaged as a secondary effect. It is necessary to clarify the underlying molecular mechanisms in future research.

It is also necessary to discuss various possibilities that may explain the results we obtained using the *Aeromonas* strains other than those with high ASP productivity. Strain 104 did not produce ASP. This strain did not decrease the TER value and did not cause bacterial translocation across the T84 monolayer after infection with T84 cells (**Figure 1**). Strain 106 also did not produce ASP. Although this strain caused a slight decrease in the TER value with a significant difference, bacterial translocation of this strain across the T84 monolayer was not observed (**Figure 1**). In addition, neither strain caused degradation of the ZO proteins or claudins after infection with T84 cells (**Figure 2**). We thus speculate that these strains are not fundamentally tissue-invasive *Aeromonas* strains.

The properties exhibited by the remaining six strains were very complex. We will first consider the four strains that showed lower ASP productivity (121, 122, 124, and 125). Although strains 122 and 125 seemed to cause the degradation of ZO proteins and claudin-7, it was not always possible to obtain data showing a significant difference in the degradation of ZO proteins and claudin-7 at 2 hr after infection with these strains (**Figure 2**). In contrast, strains 121 and 124 did not cause the degradation of ZO proteins and claudin-7 significantly at 2 hr after infection. Nevertheless, all four of these strains markedly decreased the TER value and caused bacterial translocation across the T84 monolayer (as seen in the strains with high ASP productivity) at 9 hr after infection with these strains (**Figure 1**). We therefore presumed that even low-ASP-producing strains can induce disruption of intestinal barrier function but may take longer to cause a decrease of the TER value and cause bacterial translocation. Then we further examined changes in the TER values over time using strains 121, 124, and 288. The ANOVA analysis revealed that even the strains showing low ASP productivity (such as strains 121 and 124) caused a significant decrease in the TER value 3 hr or 4 hr after infection to the apical side of the T84 monolayer (**Supplementary Figure S1**). From these results, we suspect that the tissue invasion of these four strains after infection may occur by the same strategy as that used by the strains with high ASP productivity, although it takes some time because of their low ASP productivity.

In addition, it is also possible that the progression of the decrease in the TER value may differ slightly between the strains with low ASP productivity. For example, a comparison of the time-courses of the decrease in the TER value using the strains 121 and 124 reveals that the TER value after 4 hr was significantly decreased in strain 124, whose ASP productivity was lower than that of strain 121 (**Supplementary Figure S1**). In some *Aeromonas* strains with low ASP productivity, unknown pathogenic factors other than ASP may also be involved to increase the invasiveness of the bacteria, leading to a decrease in the barrier function of the T84 monolayer. It is thus necessary to investigate the pathogenicity of the strains with low ASP productivity at the molecular level to clarify these unresolved issues.

We next consider *A. sobria* strain 101, which showed low ASP productivity and caused the degradation of ZO proteins and claudin-7 to some extent, like the four strains discussed above (**Figure 3**). Although the TER value at 9 hr post-infection of strain 101 was decreased to some degree, we did not observe bacterial translocation across the T84 monolayer. This result is inconsistent with the above-described findings. We speculate that strain 101, like the other strains with ASP productivity, can open a paracellular route to some extent but the motility of the strain may be poor, so that sufficient tissue invasion does not occur. We plan to further investigate the characteristics of this strain in a future study to explore this possibility.

On the other hand, the characteristics of the *A. sobria* strain 115 was completely different from those described above for the other 10 strains examined. Although strain 115 had no ASP productivity and did not cause any degradation of ZO proteins or claudins or a decrease in the TER value after infection with T84 cells, some bacterial translocation across the T84 monolayer was clearly observed. We speculate that strain 115 may invade tissue by another strategy. Sun et al. (2020) reported that the effector protein SpvB, which is transported from a *Salmonella* strain into intestinal epithelial cells by a type III secretion system (T3SS), causes intestinal epithelial barrier dysfunction. Like this strategy, some *Aeromonas* strains without the ability to produce ASP may have acquired a new pathogenic gene that enables the strains to invade tissue. Another possibility is that the bacteria themselves permeate the tissue through a transcellular pathway rather than a paracellular pathway, as is the case with other *Salmonella* spp. (Finlay and Falkow, 1989; Finlay and Falkow, 1990). We plan to test these hypotheses in future research.

In conclusion, our findings demonstrated that ASP produced by the *A. sobria* strains acts on protein components of TJs such as claudin-7 and ZO proteins, at least in the *A. sobria* strains with high ASP productivity. ASP also acts on protein components of AJs such as nectin-2 and afadin (Kobayashi et al., 2019). We suspect that the combination of these effects could ultimately lead to the destruction of the barrier function of the intestinal epithelium and progress to the aggravation of the infectious diseases caused by *A. sobria*.

As mentioned above, another serine protease produced by various Gram-negative bacteria, i.e., HtrA, is reported to be involved in the degradation of protein components constituting junctional complexes (e.g., occludin and claudin-8 constituting TJs, E-cadherin constituting AJs) (Sharafutdinov et al., 2020). However, the pathogenic factors that degrade nectin-2 constituting AJs and claudin-7 constituting TJs (other than ASP) are not yet known. We suspect that ASP acts on the protein components of junctional complexes with a substrate specificity that differs from the substrate specificity of other virulent factors and leads to the disruption of the intestinal barrier function. Investigations of whether an antibody against ASP completely blocks the above effects may be useful for understanding the role of ASP. We hope to clarify the remaining unsolved issues in further studies.

## Data Availability Statement

The raw data supporting the conclusions of this article will be made available by the authors, without undue reservation.

## Author Contributions

MU and HK conceived and designed the experiments. MU and HK performed the experiments and analyses. SS performed the experiments and provided supervision. ET and KO contributed to the collection of the *Aeromonas* samples. HY wrote the manuscript with contributions from all co-authors. All authors contributed to the article and approved the submitted version.

## Funding

This work was supported in part by grants-in-aid from the Japan Society for the Promotion of Science (JSPS) KAKENHI, nos. 16K18926 and 20K07504.

## Conflict of Interest

The authors declare that the research was conducted in the absence of any commercial or financial relationships that could be construed as a potential conflict of interest.

## Publisher’s Note

All claims expressed in this article are solely those of the authors and do not necessarily represent those of their affiliated organizations, or those of the publisher, the editors and the reviewers. Any product that may be evaluated in this article, or claim that may be made by its manufacturer, is not guaranteed or endorsed by the publisher.

## Supplementary Material

Supplementary Figure 1 Changes in the TER values over time after infection of *A. sobria* strains 121, 124, and 288 to the apical side of the T84 intestinal monolayer. T84 cells were grown on a Transwell system and then infected with *A. sobria* strains. After 1 hr, 2 hr, 3 hr, and 4 hr of infection (MOI = 5), the TER value was measured. The experiments were performed in triplicate. The data are mean ± SD (error bars). The data obtained were subjected to an ANOVA analysis. *p < 0.05.

## References

[B1] Abfalter C. M. Bernegger S. Jarzab M. Posselt G. Ponnuraj K. Wessler S. (2019). The Proteolytic Activity of *Listeria Monocytogenes* HtrA. BMC Microbiol. 19, 255. doi: 10.1186/s12866-019-1633-1 31726993 PMC6857308

[B2] Backert S. Bernegger S. Skórko-Glonek J. Wessler S. (2018). Extracellular HtrA Serine Proteases: An Emerging New Strategy in Bacterial Pathogenesis. Cell Microbiol. 20, e12845. doi: 10.1111/cmi.12845 29582532

[B3] Bhowmick U. D. Bhattacharjee S. (2018). Bacteriological, Clinical and Virulence Aspects of *Aeromonas-*Associated Diseases in Humans. Pol. J. Microbiol. 67, 137–149. doi: 10.21307/pjm-2018-020 30015452 PMC7256846

[B4] Borrell N. Acinas S. G. Figueras M. J. Martinez-Murcia A. J. (1997). Identification of *Aeromonas* Clinical Isolates by Restriction Fragment Length Polymorphism of PCR-Amplified 16S rRNA Genes. J. Clin. Microbiol. 35, 1671–1674. doi: 10.1128/jcm.35.7.1671-1674.1997 9196171 PMC229819

[B5] Drolia R. Bhunia A. K. (2019). Crossing the Intestinal Barrier *via Listeria* Adhesion Protein and Internalin A. Trends Microbiol. 27, 408–425. doi: 10.1016/j.tim.2018.12.007 30661918

[B6] Fanning A. S. Jameson B. J. Jesaitis L. A. Anderson J. M. (1998). The Tight Junction Protein ZO-1 Establishes a Link Between the Transmembrane Protein Occludin and the Actin Cytoskeleton. J. Biol. Chem. 273, 29745–29753. doi: 10.1074/jbc.273.45.29745 9792688

[B7] Farquhar M. G. Palade G. E. (1963). Junctional Complexes in Various Epithelia. J. Cell Biol. 17, 375–412. doi: 10.1083/jcb.17.2.375 13944428 PMC2106201

[B8] Finlay B. B. Falkow S. (1989). Common Themes in Microbial Pathogenicity. Microbiol. Rev. 53, 210–230. doi: 10.1128/mr.53.2.210-230.1989 2569162 PMC372728

[B9] Finlay B. B. Falkow S. (1990). *Salmonella* Interactions With Polarized Human Intestinal Caco-2 Epithelial Cells. J. Infect. Dis. 162, 1096–1106. doi: 10.1093/infdis/162.5.1096 2230236

[B10] Fujii Y. Nomura T. Kanzawa H. Kameyama M. Yamanaka H. Akita M.. (1998). Purification and Characterization of Enterotoxin Produced by *Aeromonas Sobria* . Microbiol. Immunol. 42, 703–714. doi: 10.1111/j.1348-0421.1998.tb02343.x 9858466

[B11] Harrer A. Bücker R. Boehm M. Zarzecka U. Tegtmeyer N. Sticht H.. (2019). *Campylobacter Jejuni* Enters Gut Epithelial Cells and Impairs Intestinal Barrier Function Through Cleavage of Occludin by Serine Protease HtrA. Gut Pathog. 11, 4. doi: 10.1186/s13099-019-0283-z 30805031 PMC6373145

[B12] Hartsock A. Nelson W. J. (2008). Adherens and Tight Junctions: Structure, Function and Connections to the Actin Cytoskeleton. Biochim. Biophys. Acta Biomembr. 1778, 660–669. doi: 10.1016/j.bbamem.2007.07.012 PMC2682436 17854762

[B13] Herz K. Vimont S. Padan E. Berche P. (2003). Roles of NhaA, NhaB, and NhaD Na+/H+ Antiporters in Survival of *Vibrio Cholerae* in a Saline Environment. J. Bacteriol. 185, 1236–1244. doi: 10.1128/jb.185.4.1236-1244.2003 12562793 PMC142861

[B14] Huber P. (2020). Targeting of the Apical Junctional Complex by Bacterial Pathogens. Biochim. Biophys. Acta Biomembr. 1862, 183237. doi: 10.1016/j.bbamem.2020.183237 32126234

[B15] Hutchinson L. E. Franke J. D. Mailey B. A. (2021). Necrotizing Fasciitis Secondary to Lake Water Inoculation With *Aeromonas Sobria*: A Case Report. Medicine 100, e24981. doi: 10.1097/md.0000000000024981 33725868 PMC7969264

[B16] Imamura T. Kobayashi H. Khan R. Nitta H. Okamoto K. (2006). Induction of Vascular Leakage and Blood Pressure Lowering Through Kinin Release by a Serine Proteinase From *Aeromonas Sobria* . J. Immunol. 177, 8723–8729. doi: 10.4049/jimmunol.177.12.8723 17142774

[B17] Imamura T. Nitta H. Wada Y. Kobayashi H. Okamoto K. (2008). Impaired Plasma Clottability Induction Through Fibrinogen Degradation by ASP, a Serine Protease Released From *Aeromonas Sobria* . FEMS Microbiol. Lett. 284, 35–42. doi: 10.1111/j.1574-6968.2008.01184.x 18462393 PMC2613230

[B18] Itoh M. Nagafuchi A. Moroi S. Tsukita S. (1997). Involvement of ZO-1 in Cadherin-Based Cell Adhesion Through Its Direct Binding to α Catenin and Actin Filaments. J. Cell Biol. 138, 181–192. doi: 10.1083/jcb.138.1.181 9214391 PMC2139940

[B19] Itoh M. Tsukita S. Yamazaki Y. Sugimoto H. (2012). Rho GTP Exchange Factor ARHGEF11 Regulates the Integrity of Epithelial Junctions by Connecting ZO-1 and RhoA-Myosin II Signaling. Proc. Natl. Acad. Sci. 109, 9905–9910. doi: 10.1073/pnas.1115063109 22665792 PMC3382488

[B20] Janda J. M. Abbott S. L. (1998). Evolving Concepts Regarding the Genus *Aeromonas*: An Expanding Panorama of Species, Disease Presentations, and Unanswered Questions. Clin. Infect. Dis. 27, 332–344. doi: 10.1086/514652 9709884

[B21] Janda J. M. Abbott S. L. (2010). The Genus Aeromonas: Taxonomy, Pathogenicity, and Infection. Clin. Microbiol. Rev. 23, 35–73. doi: 10.1128/cmr.00039-09 20065325 PMC2806660

[B22] Janda J. M. Brenden R. (1987). Importance of *Aeromonas Sobria* in *Aeromonas* Bacteremia. J. Infect. Dis. 155, 589–591. doi: 10.1093/infdis/155.3.589 3805781

[B23] Kobayashi H. Seike S. Yamaguchi M. Ueda M. Takahashi E. Okamoto K.. (2019). *Aeromonas Sobria* Serine Protease Decreases Epithelial Barrier Function in T84 Cells and Accelerates Bacterial Translocation Across the T84 Monolayer *In Vitro* . PloS One 14, e0221344. doi: 10.1371/journal.pone.0221344 31419250 PMC6697317

[B24] Kobayashi H. Utsunomiya H. Yamanaka H. Sei Y. Katunuma N. Okamoto K.. (2009). Structural Basis for the Kexin-Like Serine Protease From *Aeromonas Sobria* as Sepsis-Causing Factor. J. Biol. Chem. 284, 27655–27663. doi: 10.1074/jbc.m109.006114 19654332 PMC2785694

[B25] Kobayashi H. Yoshida T. Miyakawa T. Tashiro M. Okamoto K. Yamanaka H.. (2015). Structural Basis for Action of the External Chaperone for a Propeptide-Deficient Serine Protease From *Aeromonas Sobria* . J. Biol. Chem. 290, 11130–11143. doi: 10.1074/jbc.m114.622852 25784551 PMC4409271

[B26] Kuroda T. Mizushima T. Tsuchiya T. (2005). Physiological Roles of Three Na+/H+ Antiporters in the Halophilic Bacterium *Vibrio Parahaemolyticus* . Microbiol. Immunol. 49, 711–719. doi: 10.1111/j.1348-0421.2005.tb03662.x 16113500

[B27] Leibowitz M. J. Wickner R. B. (1976). A Chromosomal Gene Required for Killer Plasmid Expression, Mating, and Spore Maturation in *Saccharomyces Cerevisiae* . Proc. Natl. Acad. Sci. U.S.A. 73, 2061–2065. doi: 10.1073/pnas.73.6.2061 778853 PMC430448

[B28] Nakao Y. Nakamura S. Mikami S. Kishimoto T. Uraoka N. Sakamoto N.. (2016). A Case of *Aeromonas Sobria* Sepsis With Skin and Soft Tissue Infection. Anesthesia Resuscitation 52, 3–6.

[B29] Nishibuchi M. Kumagai K. Kaper J. B. (1991). Contribution of the *Tdh*1 Gene of Kanagawa Phenomenon-Positive *Vibrio Parahaemolyticus* to Production of Extracellular Thermostable Direct Hemolysin. Microb. Pathogen. 11, 453–460. doi: 10.1016/0882-4010(91)90042-9 1795634

[B30] Nitta H. Imamura T. Wada Y. Irie A. Kobayashi H. Okamoto K.. (2008). Production of C5a by ASP, a Serine Protease Released From *Aeromonas Sobria* . J. Immunol. 181, 3602–3608. doi: 10.4049/jimmunol.181.5.3602 18714034

[B31] Nitta H. Kobayashi H. Irie A. Baba H. Okamoto K. Imamura T. (2007). Activation of Prothrombin by ASP, a Serine Protease Released From *Aeromonas Sobria* . FEBS Lett. 581, 5935–5939. doi: 10.1016/j.febslet.2007.11.076 18067862

[B32] Okamoto K. Nomura T. Hamada M. Fukuda T. Noguchi Y. Fujii Y. (2000). Production of Serine Protease of *Aeromonas Sobria* Is Controlled by the Protein Encoded by the Gene Lying Adjacent to the 3' End of the Protease Gene. Microbiol. Immunol. 44, 787–798. doi: 10.1111/j.1348-0421.2000.tb02565.x 11092244

[B33] Pemberton J. M. Kidd S. P. Schmidt R. (1997). Secreted Enzymes of *Aeromonas* . FEMS Microbiol. Lett. 152, 1–10. doi: 10.1111/j.1574-6968.1997.tb10401.x 9228763

[B34] Radhakrishnan D. Mc A. Hutterer E. Wessler S. Ponnuraj K. (2021). High Temperature Requirement A (HtrA) Protease of *Listeria Monocytogenes* and its Interaction With Extracellular Matrix Molecules. FEMS Microbiol. Lett. 368, nab141. doi: 10.1093/femsle/fnab141 34755852

[B35] Rosenthal R. Heydt M. S. Amasheh M. Stein C. Fromm M. Amasheh S. (2012). Analysis of Absorption Enhancers in Epithelial Cell Models. Ann. NY Acad. Sci. 1258, 86–92. doi: 10.1111/j.1749-6632.2012.06562.x 22731720

[B36] Schmidt T. P. Perna A. M. Fugmann T. Böhm M. Hiss J. Haller S.. (2016). Identification of E-Cadherin Signature Motifs Functioning as Cleavage Sites for *Helicobacter Pylori* HtrA. Sci. Rep-UK 6, 23264. doi: 10.1038/srep23264 PMC4794652 26983597

[B37] Schweizer H. P. Hoang T. T. (1995). An Improved System for Gene Replacement and xylE Fusion Analysis in *Pseudomonas Aeruginosa* . Gene 158, 15–22. doi: 10.1016/0378-1119(95)00055-b 7789804

[B38] Sharafutdinov I. Esmaeili D. S. Harrer A. Tegtmeyer N. Sticht H. Backert S. (2020). *Campylobacter Jejuni* Serine Protease HtrA Cleaves the Tight Junction Component Claudin-8. Front. Cell Infect. Mi 10. doi: 10.3389/fcimb.2020.590186 PMC7752809 33364202

[B39] Shimoji N. Murayama M. Furuno Y. Uechi A. Tamaki I. Tedokon M.. (2015). Basic Analysis in the Case of *Aeromonas* Bacteria Detected in Areas Other Than the Intestinal Tract. Japanese J. Med. Technol. 64, 295–301. doi: 10.14932/jamt.14-93

[B40] Song P. Deng J. Hou T. Fu X. Zhang L. Sun L.. (2019). *Aeromonas Sobria* Peritonitis in a Peritoneal Dialysis (PD) Patient: A Case Report and Review of the Literature. BMC Nephrol. 20, 180. doi: 10.1186/s12882-019-1361-7 31109291 PMC6528350

[B41] Spadaro S. Berselli A. Marangoni E. Romanello A. Colamussi M. V. Ragazzi R.. (2014). *Aeromonas Sobria* Necrotizing Fasciitis and Sepsis in an Immunocompromised Patient: A Case Report and Review of the Literature. J. Med. Case Rep. 8, 315. doi: 10.1186/1752-1947-8-315 25245365 PMC4177370

[B42] Steed E. Balda M. S. Matter K. (2010). Dynamics and Functions of Tight Junctions. Trends Cell Biol. 20, 142–149. doi: 10.1016/j.tcb.2009.12.002 20061152

[B43] Sun L. Yang S. Deng Q. Dong K. Li Y. Wu S.. (2020). *Salmonella* Effector SpvB Disrupts Intestinal Epithelial Barrier Integrity for Bacterial Translocation. Front. Cell Infect. Mi 10. doi: 10.3389/fcimb.2020.606541 PMC7773751 33392110

[B44] Takai Y. Ikeda W. Ogita H. Rikitake Y. (2008a). The Immunoglobulin-Like Cell Adhesion Molecule Nectin and Its Associated Protein Afadin. Annu. Rev. Cell Dev. Biol. 24, 309–342. doi: 10.1146/annurev.cellbio.24.110707.175339 18593353

[B45] Takai Y. Miyoshi J. Ikeda W. Ogita H. (2008b). Nectins and Nectin-Like Molecules: Roles in Contact Inhibition of Cell Movement and Proliferation. Nat. Rev. Mol. Cell Biol. 9, 603–615. doi: 10.1038/nrm2457 18648374

[B46] Umeda K. Ikenouchi J. Katahira-Tayama S. Furuse K. Sasaki H. Nakayama M.. (2006). ZO-1 and ZO-2 Independently Determine Where Claudins Are Polymerized in Tight-Junction Strand Formation. Cell 126, 741–754. doi: 10.1016/j.cell.2006.06.043 16923393

[B47] Zihni C. Balda M. S. Matter K. (2014). Signalling at Tight Junctions During Epithelial Differentiation and Microbial Pathogenesis. J. Cell Sci. 127, 3401–3413. doi: 10.1242/jcs.145029 25125573

