# Supplementary figures and images for: Aeromonas sobria Serine Protease Degrades Several Protein Components of Tight Junctions and Assists Bacterial Translocation Across the T84 Monolayer
Source: Front Cell Infect Microbiol. 2022 Feb 22;12:824547. doi: 10.3389/fcimb.2022.824547 (PMC8902146; doi:10.3389/fcimb.2022.824547)

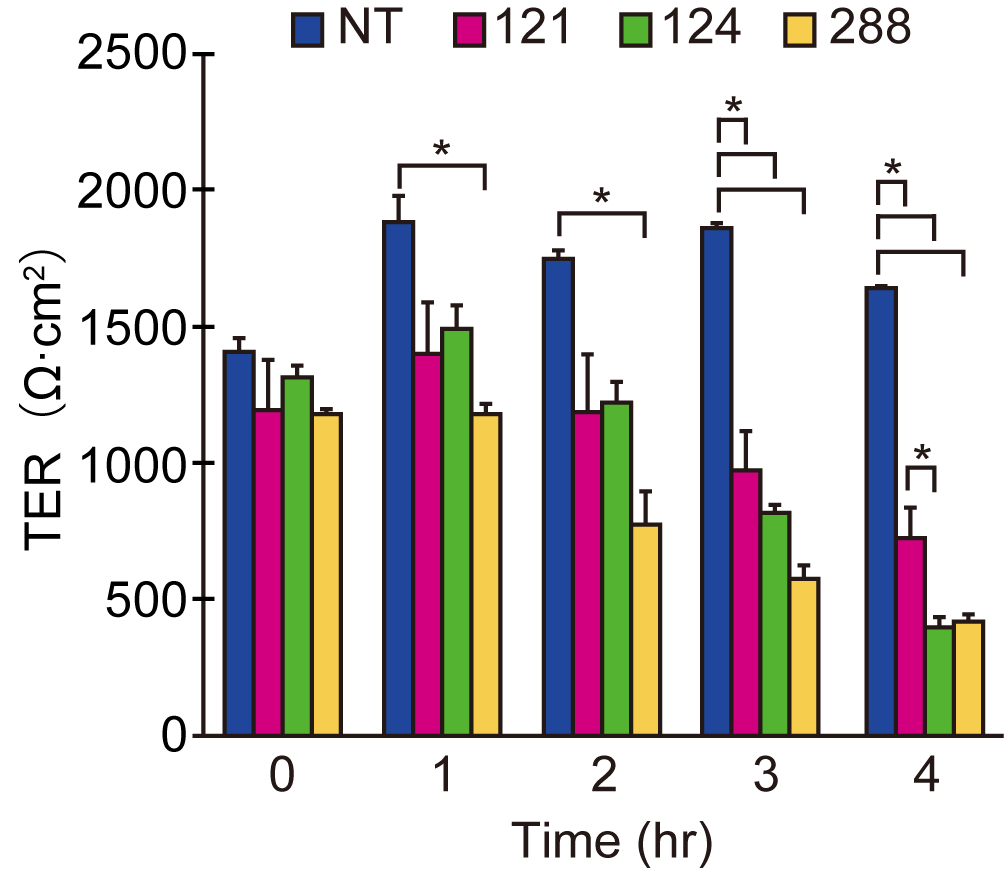

Supplement: Supplementary Figure 1 — Changes in the TER values over time after infection of A. sobria strains 121, 124, and 288 to the apical side of the T84 intestinal monolayer. T84 cells were grown on a Transwell system and then infected with A. sobria strains. After 1 hr, 2 hr, 3 hr, and 4 hr of infection (MOI = 5), the TER value was measured. The experiments were performed in triplicate. The data are mean ± SD (error bars). The data obtained were subjected to an ANOVA analysis. *p < 0.05. [file Image_1.tif]
